# Numerical Analysis of Residual Stress in Swing-Arc Narrow-Gap Gas Metal Arc Welding

**DOI:** 10.3390/ma18040803

**Published:** 2025-02-12

**Authors:** Yejun Wu, Jiayou Wang, Guoxiang Xu, Yuqin Jiang

**Affiliations:** 1 School of Intelligent Manufacturing, Changzhou Vocational Institute of Engineering, Changzhou 213164, China; wuyejun1985@126.com; 2 Jiangsu Provincial Key Laboratory of Advanced Welding Technology, Jiangsu University of Science and Technology, Zhenjiang 212003, China; jywang@just.edu.cn (J.W.); jyuqing2012@126.com (Y.J.)

**Keywords:** narrow gap of swing arc, numerical simulation, residual stress, ANSYS

## Abstract

In order to gain a deeper understanding of the distribution of residual stresses in swing-arc narrow-gap GMA welding, this paper comprehensively considers the arc motion trajectory and joint geometry and establishes a three-dimensional finite element numerical analysis model for residual stresses based on elastic–plastic theory. Using the Ansys software, the welding residual stresses were calculated under swing frequencies of 4 Hz, 3 Hz, and 2 Hz, and the distribution characteristics of residual stresses were analyzed. The results indicate that the model effectively and accurately represents the movement trajectory and distribution characteristics of the swing arc. Furthermore, the calculated temperature field and residual stress outcomes align closely with the experimental findings, thereby validating the accuracy of the model. Under varying swing frequencies, the distribution patterns of residual stress along each sampling line exhibit a consistent similarity. The residual stress is predominantly concentrated in the weld zone and the adjacent heat-affected zone, while it remains relatively low in areas further away from the weld. As the swing frequency increases, the residual stress decreases. The reason for this is that an increase in swing frequency can lead to a more uniform distribution of arc heat within the weld bead, ultimately resulting in lower residual stress.

## 1. Introduction

Swing-arc narrow-gap gas metal arc (GMA) welding, characterized by its high efficiency, superior quality, and economic advantages, has emerged as an ideal technology for the welding of thick plates [1,2,3,4]. Currently, research on swing-arc narrow-gap GMA welding primarily emphasizes equipment and process aspects, while investigations into its underlying physical mechanisms remain relatively limited. Jiayou Wang [5] studied the effects of different swing frequencies, sidewall dwell times, and reserved gaps on weld formation in swing-arc narrow-gap GMA welding. The results showed that, with an increasing swing frequency and sidewall dwell time and a decreasing reserved gap, the sidewall penetration and twin peaks at the bottom of the bead are enhanced. Wenji Liu et al. [6] successfully achieved seam tracking in narrow-gap GMA welding with a swing arc by extracting deviations through the integration of multiple statistical features derived from current information within the swing period. Currently, the research on narrow-gap GMA welding with a swing arc primarily emphasizes the welding processes and equipment, which hampers a comprehensive understanding of the underlying physical phenomena. However, with the ongoing advancements in computer technology and numerical analysis methods, numerical simulation has emerged as a crucial tool for investigating these physical processes [7,8,9,10,11]. Shuai Yuan et al. [12] modeled and calculated the temperature field of the narrow-gap weld with swing-arc MAG welding using a double-ellipsoid heat source and analyzed the distribution characteristics of the temperature field. Guoxiang Xu et al. [13,14] established a three-dimensional model of the fluid flow in the molten pool of narrow-gap GMAW vertical welding with a swing arc, which is applicable to the solid wire process. A.S. Elmesalamy et al. [15] used finite element modeling to study the influence of the thermal cycle curve on the residual stress of laser narrow-gap welding. Wenhang Li et al. [10] conducted a finite element analysis of the multi-layer rotating-arc narrow-gap MAG welding of medium and thick plates. Based on the thermal equivalent and welding characteristics, four-point heat sources and two equivalent heat sources were proposed and compared. The residual stress and deformation were calculated based on the temperature field under four different welding conditions. Guangkai Zhang [16] conducted numerical simulations on the stress field of multi-layer swing-arc narrow-gap GMA welding, comparing and analyzing the residual stresses under two welding conditions of swing and non-swing, but there is no research on the influence of different welding parameters on the residual stress. This paper comprehensively considers the arc swing trajectory, joint geometric characteristics, etc., and establishes a numerical analysis model for the stress field of swing-arc narrow-gap GMA welding. Compared with ordinary narrow-gap GMA welding, this model has a more complex moving path of the heat source, higher computational difficulty, and higher novelty. This paper uses the ANSYS version 19.0 software to calculate and analyze the welding residual stress of workpieces, providing a theoretical basis for a deeper understanding of the stress distribution of the workpiece in swing-arc narrow-gap GMA welding, optimizing the welding process and improving the equipment. This paper also measures the residual stress of the joint using the blind hole method and obtains the joint cross section through macroscopic metallography to verify the accuracy of the model.

## 2. Experimental Section

**Figure 1:**
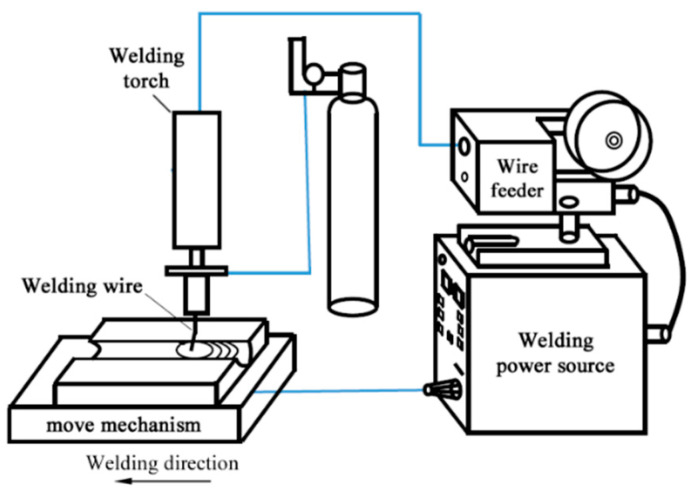
Schematic diagram of the principle of the swing-arc narrow-gap GMA welding system.

**Figure 2:**
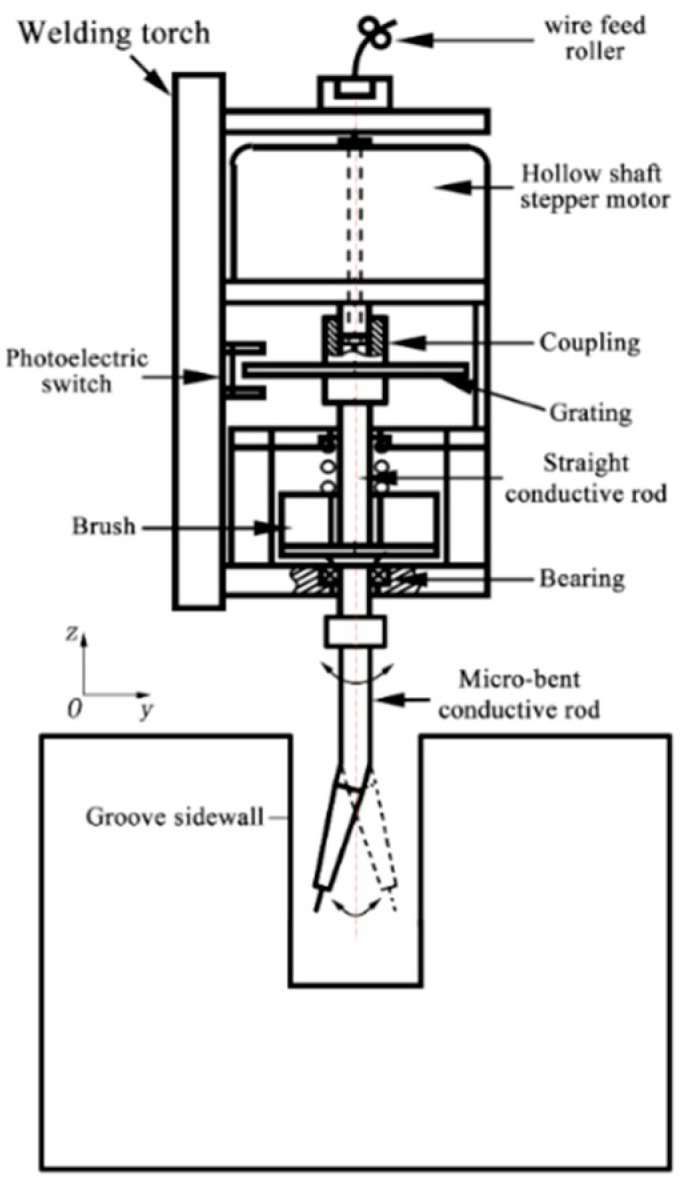
Schematic diagram of the structure principle of the oscillating-arc narrow-gap GMA welding torch.

**Figure 3:**
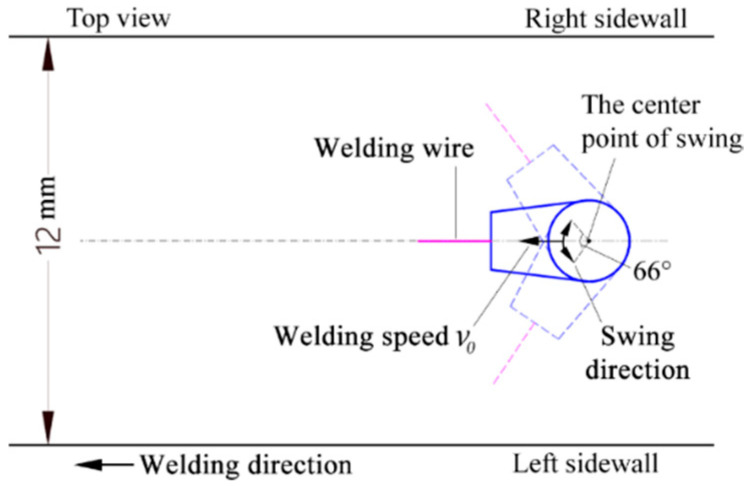
Diagram of wire swing.

**Figure 4:**
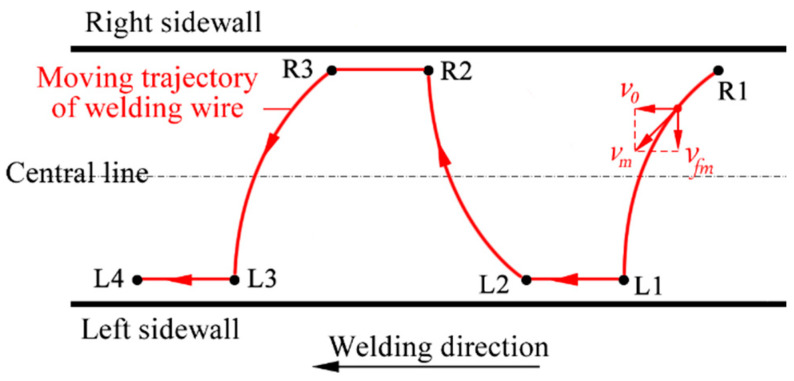
Moving trajectory of welding wire.

Figure 1 illustrates the schematic diagram of the swing-arc narrow-gap GMA welding system examined in this study. Figure 2 depicts the structural schematic of the swing-arc narrow-gap GMA welding torch. During the welding process, a slightly curved conductive rod rotates laterally around its central axis, which causes the end of the main wire to swing back and forth within a specified angular range. Concurrently, the welding torch advances continuously at a predetermined speed $v_{0}$ along the direction of welding. Figure 3 and Figure 4 present the movement trajectory of the welding wire. After swinging from point R1 on the right to point L1 on the left, the wire ceases its swing and moves at speed $v_{0}$ towards point L2 along the weld path for a duration to enhance the sidewall penetration; it then resumes swinging towards point R2 where it remains stationary momentarily. This sequence is repeated continuously.

**Figure 5:**
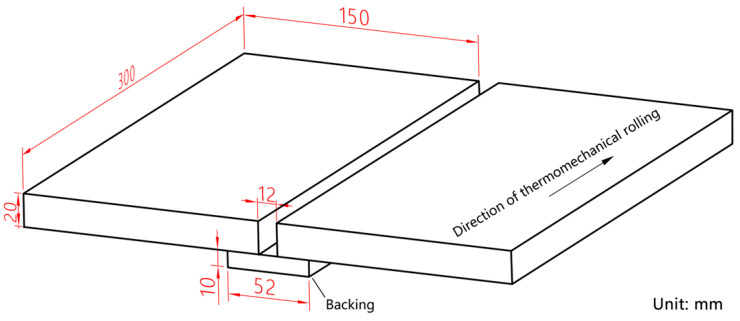
Groove type of welding.

Through preliminary process experiments, a well-formed joint can be obtained when the current is set to 320 A, the arc voltage is 30 V, and the welding speed $v_{0}$ is 240 mm/min. Therefore, this article adopts the above welding process parameters. The swing angle is maintained at 66°, while the bending angle of the conductive rod is specified as 8°. The swing frequencies utilized are 2 Hz, 3 Hz, and 4 Hz, accompanied by a dwell time on the sidewall of 60 ms. The base material employed in this study is Q370qE, supplied in a TMCP + T state (thermomechanical controlled processing followed by tempering). The wire grade used for welding is ER80S-G with a diameter of Φ1.2 mm. Additionally, the shielding gas composition consists of 80% Ar and 20% CO_2_. It should be noted that the material used for the welding backing matches that of the base material. Figure 5 illustrates both the form and dimensions of the welding groove.

**Figure 6:**
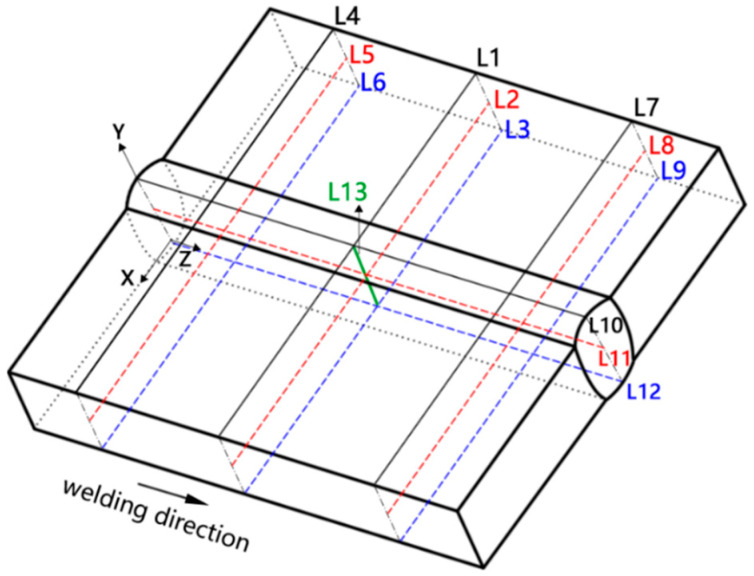
Schematic diagram of sampling lines for residual stress distribution.

To facilitate a more comprehensive analysis of the distribution of residual stress within the workpiece, this paper presents an extraction and comparison of residual stress measurements taken from multiple positions on the workpiece. Specifically, sampling lines L1, L2, and L3 were positioned at the upper surface, middle section, and lower surface of the central region of the workpiece (*z* = 150 mm). Sampling lines L4, L5, and L6 corresponded to the upper surface, middle section, and lower surface at the head position of the workpiece (*z* = 10 mm). Similarly, sampling lines L7, L8, and L9 were located at these same respective positions for the tail end of the workpiece (*z* = 290 mm). Additionally, sampling lines L10, L11, and L12 were situated at equivalent locations along both surfaces in relation to the midpoint of the weld seam (*x* = 0 mm), while sampling line L13 was placed at mid-weld seam coordinates (*x* = 0 mm and *z* = 150 mm) extending through its thickness direction, as illustrated in Figure 6.

## 3. Finite Element Modeling

The residual stress associated with swing-arc narrow-gap GMA welding was simulated using a three-dimensional finite element model based on thermoelastic–plastic principles. To enhance computational efficiency, an uncoupled thermomechanical formulation [17,18,19] was employed in the analysis. Initially, transient heat conduction analysis was performed to compute the welding temperature profile and its history, which is independent from the stress analysis; subsequently, this temperature profile served as a thermal load for determining the residual stress. In this study, the ANSYS software was utilized to compute the residual stresses resulting from the welding process.

### 3.1. Thermal Analysis

#### 3.1.1. Heat Source Model

Given the significant computational cost associated with residual stress analysis, researchers have proposed an efficient subsection heat source technology to enhance calculation efficiency. The accuracy of this model has been validated, and it is now widely utilized in various applications [20,21,22]. Consequently, this paper adopts a uniform-body segmented heat source to characterize the action characteristics of the heat source. The heat flux density function for the uniform-body heat source can be expressed by the following equation [23]:

(1) $$q=\frac{\eta IU}{V}$$

In the formula, *η* denotes the thermal efficiency of welding, *I* represents the welding current, *U* signifies the voltage, and *V* indicates the volume influenced by the heat source.

#### 3.1.2. Heat Conduction Equation

Fluid flow significantly influences both the heat transfer within the weld pool and the geometry of the weld seam. However, numerical simulations of fluid flow are inherently complex. To simplify this model, only transient heat conduction is considered in the calculations, while fluid flow effects are neglected. To account for the impact of fluid flow on the temperature field of the welding molten pool, an approach involving the artificial enhancement of thermal conductivity is employed at temperatures exceeding the melting point of the base material [17]. The heat conduction equation utilized in this thermal analysis can be expressed as follows:

(2) $$\rho c\frac{\partial T}{\partial t}=\frac{\partial }{\partial x}\left(k\frac{\partial T}{\partial x}\right)+\frac{\partial }{\partial y}\left(k\frac{\partial T}{\partial y}\right)+\frac{\partial }{\partial z}\left(k\frac{\partial T}{\partial z}\right)+S_{H}$$

In the equation, T denotes temperature, ρ signifies density, c represents specific heat capacity, k indicates thermal conductivity, and S_H refers to the heat source term, which corresponds to the power density of the hybrid welding heat source.

This paper considers all surface boundary conditions while accounting for heat loss due to convection, radiation, and evaporation. The calculation formula is as follows:

(3) $$-k\frac{\partial T}{\partial \vec{n}}=-\alpha_{cr}\left(T-T_{\infty }\right)-m_{er}L_{b}$$

In the equation, $\vec{n}$ represents the unit normal vector to the surface, $\alpha_{cr}$ denotes the combined convective and radiative heat transfer coefficient, $m_{er}$ indicates the evaporation rate, $L_{b}$ signifies the latent heat of evaporation, and $T_{\infty }$ refers to the ambient temperature.

#### 3.1.3. The Trajectory of the Heat Source

**Figure 7:**
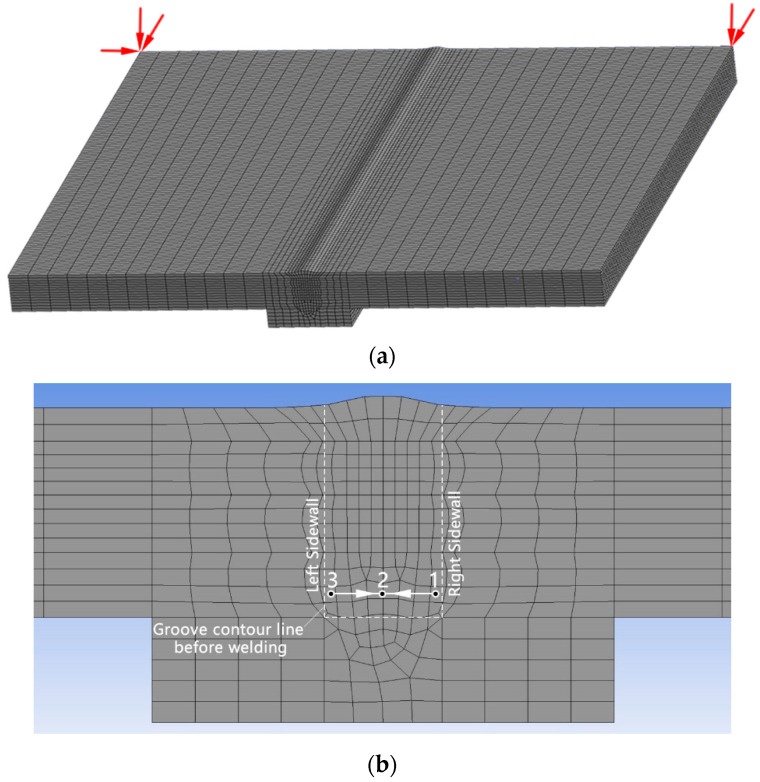
Finite element mesh: (**a**) overall mesh and (**b**) local mesh.

As previously mentioned, the welding wire not only advances along the welding direction but also swings laterally in a perpendicular manner to this direction. In accordance with the characteristics of heat source movement, subsection heat source technology is employed, segmenting the overall movement path into several sections, each treated as an equivalent heat source. As illustrated in Figure 7b, the uniform-body heat source initially moves from the right side of the weld bead (position 1) to the left, traverses through the center of the weld bead (position 2), reaches the left side of the weld bead (position 3), and remains there for a duration of 60 ms. Subsequently, it returns to its original position on the right side of the weld bead (position 1), where it again stays for another 60 ms. The heating process associated with oscillating-arc narrow-gap GMA welding is characterized by this reciprocating motion and dwell time of the heat source.

### 3.2. Mechanical Analysis

In the case of low-alloy steel, the alterations in material volume and mechanical properties resulting from solid-state transformations exert a negligible influence on the residual stress [24]. Consequently, the total strain increment can be delineated into three distinct components:

(4) $$\Delta \varepsilon =\Delta \varepsilon^{E}+{\Delta \varepsilon }^{P}+\Delta \varepsilon^{T}$$

where $\Delta \varepsilon^{E}$, ${\Delta \varepsilon }^{P}$, and $\Delta \varepsilon^{T}$ are the elastic, plastic, and thermal strain increments, respectively.

In the context of thermal and mechanical analysis, a hexahedron element was used, as shown in Figure 7. Mesh convergence was used to determine the mesh size. The mesh of the weld is the most dense, with a size of 1.2–1.7 mm, followed by the HAZ with a mesh size of 2.9–3.7 mm. During mechanical analysis, it is essential to convert thermal elements into structural elements while simultaneously applying mechanical constraints to prevent the rigid body motion of the workpiece, as illustrated in Figure 7a. The birth-and-death element method is utilized to manage the elements within the weld zone.

### 3.3. Material Properties

**Figure 8:**
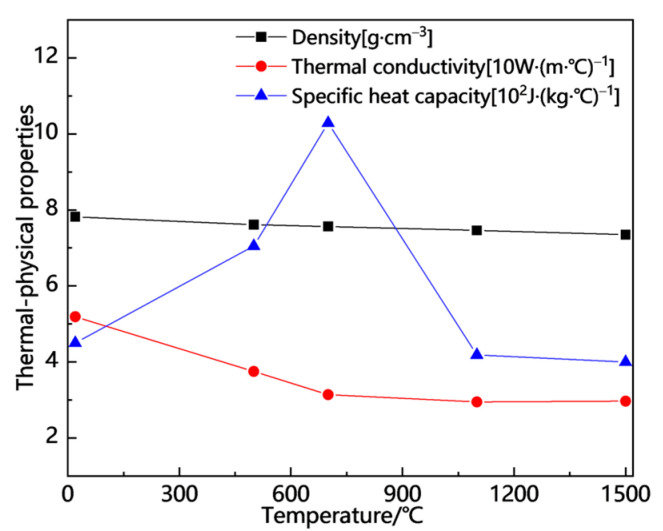
Temperature-dependent thermal–physical properties.

**Figure 9:**
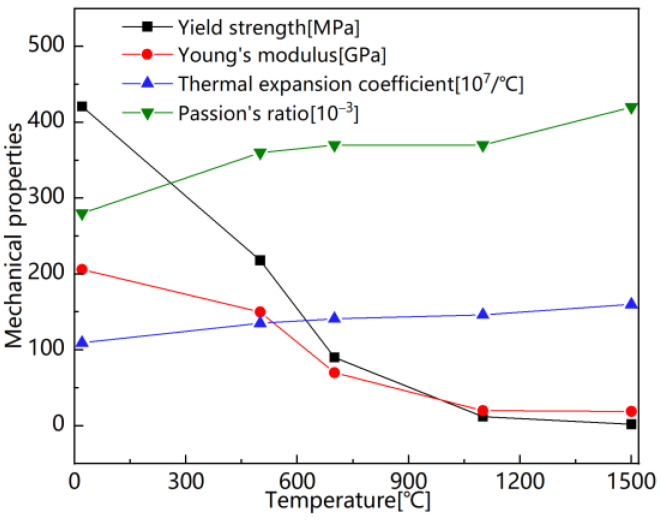
Temperature-dependent mechanical properties.

Currently, there is a limited amount of research on the welding simulation of Q370qE steel, and data regarding the material’s high-temperature performance are insufficient. This paper references the high-temperature performance data of Q355D steel, which has a similar chemical composition [25,26], and employs linear interpolation to derive the missing high-temperature performance data. The temperature-dependent thermophysical and mechanical properties utilized in this study are illustrated in Figure 8 and Figure 9.

In the present analysis, it is assumed that the elastic behavior of the welding material adheres to the isotropic Hooke’s law, while the plastic behavior is characterized by a rate-independent plasticity model. The yielding process of the workpiece is simulated in accordance with the von Mises yield criterion, and strain hardening effects are incorporated using a bilinear isotropic hardening rule.

## 4. Verification of the Model

This paper uses the blind hole method [27,28] to measure the transverse residual stress and longitudinal residual stress of the workpiece and compares them with the calculated results. This paper obtained the macroscopic morphology of the weld cross section through a microscope and compared it with the calculated temperature field. The accuracy of the calculation results was verified through the above two methods.

**Figure 10:**
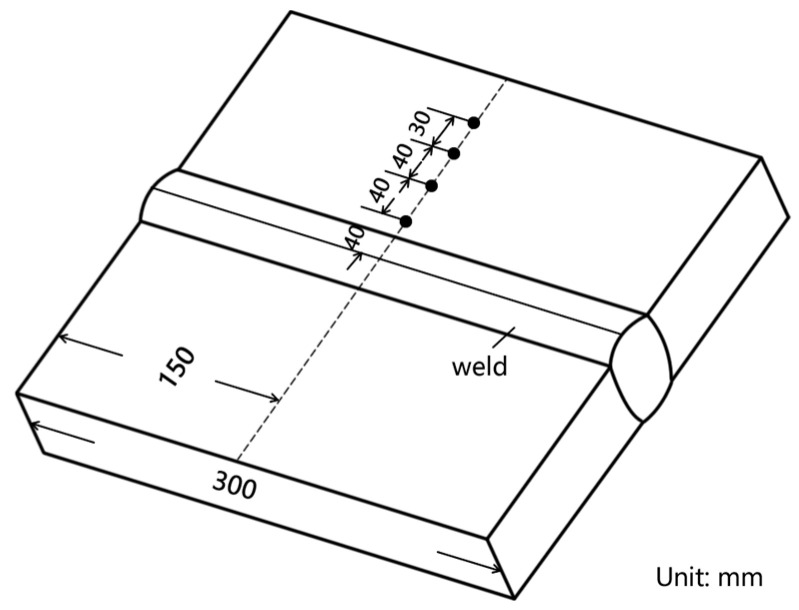
Schematic diagram of sampling points for residual stress measurement.

**Figure 11:**
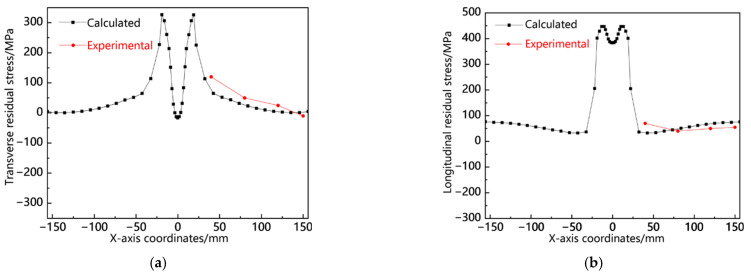
Comparison between experimental residual stress and calculation results (*f* = 3 Hz): (**a**) transverse residual stress and (**b**) longitudinal residual stress.

Using the blind hole method to measure residual stress, first, the surface of the workpiece is cleaned, the strain gauge is pasted at the position to be measured, the strain gauge lead is connected to the terminal with an electric soldering iron, and one end of the terminal is connected to the instrument lead, and the other end of the instrument lead is connected to the corresponding socket of the instrument. The compensation plate is connected in the same way. The relevant parameters are set based on the tested material and strain gauge type; then, preheating, zeroing, and drilling are performed, and the displacement and strain generated are recorded. Equations (5)–(7) [29] are solved. The specific sampling points are shown in Figure 10 and then compared with the calculated results, as shown in Figure 11. It can be seen from the figure that the simulated residual stress values are basically consistent with the measured values in terms of size and distribution, indicating the accuracy of the stress field model.

(5) $$\sigma_{1}=\frac{1}{4A}\left(\varepsilon_{1}+\varepsilon_{3}\right)-\frac{1}{4B}\sqrt{{\left(\varepsilon_{1}-\varepsilon_{2}\right)}^{2}+{\left(2\varepsilon_{2}-\varepsilon_{1}-\varepsilon_{3}\right)}^{2}}$$

(6) $$\sigma_{2}=\frac{1}{4A}\left(\varepsilon_{1}+\varepsilon_{3}\right)+\frac{1}{4B}\sqrt{{\left(\varepsilon_{1}-\varepsilon_{2}\right)}^{2}+{\left(2\varepsilon_{2}-\varepsilon_{1}-\varepsilon_{3}\right)}^{2}}$$

(7) $$\tan2\theta =\frac{2\varepsilon_{2}-\varepsilon_{1}-\varepsilon_{3}}{\varepsilon_{1}+\varepsilon_{3}}$$

**Figure 12:**
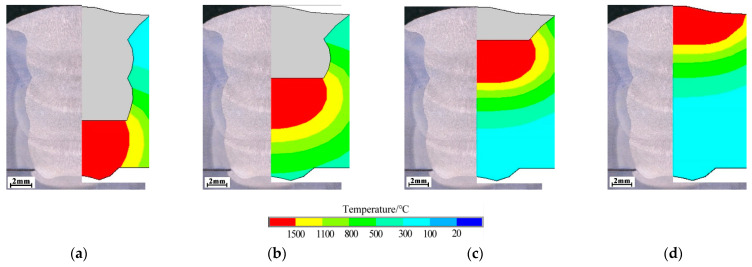
Comparison of temperature field and weld cross section (*f* = 4 Hz): (**a**) the 1st layer; (**b**) the 2nd layer; (**c**) the 3rd layer; and (**d**) the 4th layer.

Figure 12 compares the calculated shape and size of the weld cross section with the experimental results, and both are in general agreement, indicating that the developed heat source model is suitable to calculate the temperature field in welding.

## 5. Results and Discussion

### 5.1. Residual Stress Analysis with a Swing Frequency of 3 Hz

**Figure 13:**
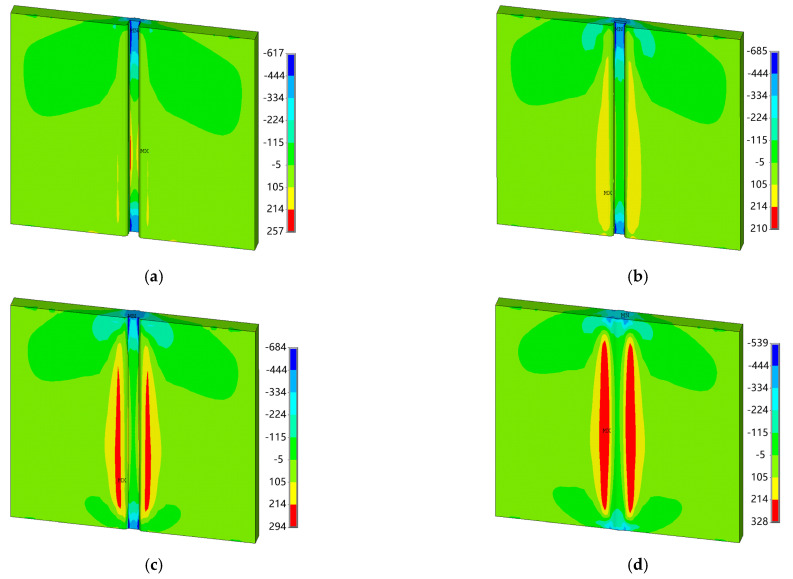
Transverse stress distribution of each weld layer (*f* = 3 Hz, MPa): (**a**) the 1st layer; (**b**) the 2nd layer; (**c**) the 3rd layer; and (**d**) the 4th layer.

Under the welding conditions described in this paper, a total of four layers are required for the workpiece to be welded. Figure 13 presents the lateral residual stress distribution diagrams for each layer of welds at a swing frequency of 3 Hz. It is observed that after welding the first layer, significant transverse compressive stresses develop at the weld toes located at both the starting and ending points, while substantial transverse tensile stresses arise at the weld toes situated in the middle section of the weld. Following the completion of the second layer, large lateral compressive stresses persist at both ends; however, there is a reduction in lateral tensile stresses at mid-weld toe locations, accompanied by an emergence of notable lateral tensile stress on the upper surface of the test plate. Upon welding the third layer, there is a decrease in lateral compressive stresses at both end points, while an increase occurs in lateral tensile stress on the upper surface near the mid-weld positions. After applying all four layers, it can be concluded that although relatively small lateral compressive stress remains evident within the weld area itself, adjacent regions exhibit significantly larger levels of lateral tensile stress. Consequently, as additional welding layers are applied, there is a discernible increasing trend in lateral tensile stress within close proximity to the mid-weld zones on the test plate. This is because the near-seam area is an HAZ; when welding the first layer, the arc has little effect on the near-seam area on the upper surface, and there is no significant phase transition in this area. However, when welding the last layer, the temperature in the near-seam area reaches the phase transition temperature, leading to grain growth, increased hardness, and increased transverse tensile stress. The greater the cooling rate, the more drastic this change will be. Conversely, a decreasing trend is noted for laterally compressive stresses present at both starting and ending points.

**Figure 14:**
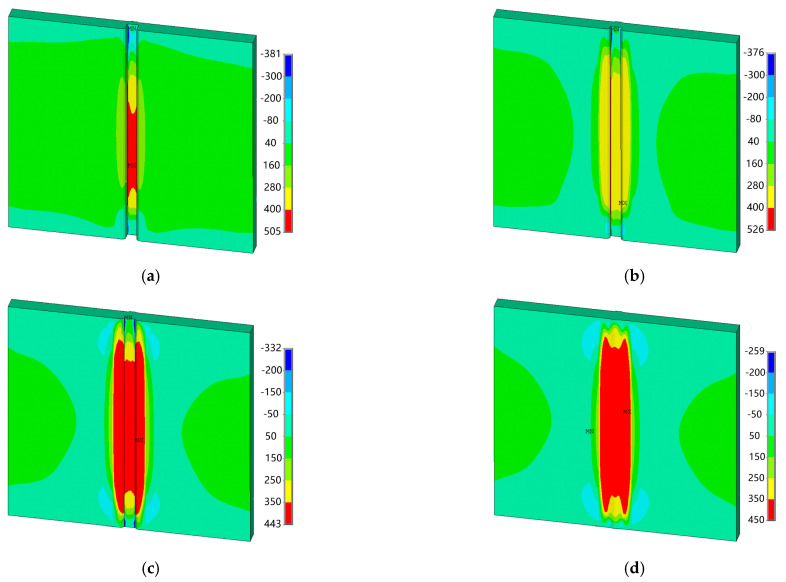
Longitudinal stress distribution of each weld layer (*f* = 3 Hz, MPa): (**a**) the 1st layer; (**b**) the 2nd layer; (**c**) the 3rd layer; and (**d**) the 4th layer.

The longitudinal residual stress distribution cloud diagrams for each layer of welds are presented in Figure 14. It is evident that after the completion of the first welding layer, significant longitudinal compressive stresses were generated at both the starting and ending weld toes, while substantial longitudinal tensile stresses emerged in the central region of the weld as well as at the weld toes. Following the second layer of welding, there was a reduction in longitudinal compressive stresses at both ends of the weld toes, alongside a decrease in longitudinal tensile stresses within the middle section; however, considerable longitudinal tensile stresses persisted at the mid-weld toes. After applying a third layer of welding, further reductions in longitudinal compressive stresses were observed at both the starting and ending weld toes, with large longitudinal tensile stresses developing in both the center of the weld and its heat-affected zone (HAZ). By completing a fourth layer of welding, it was noted that some degree of longitudinal tensile stress appeared in the middle section of the test plate’s weld, while relatively high levels remained present within the HAZ. This analysis indicates that, similar to transverse tensile stress distributions, significant concentrations of longitudinal tensile stress primarily occur near and within areas adjacent to welded joints.

**Figure 15:**
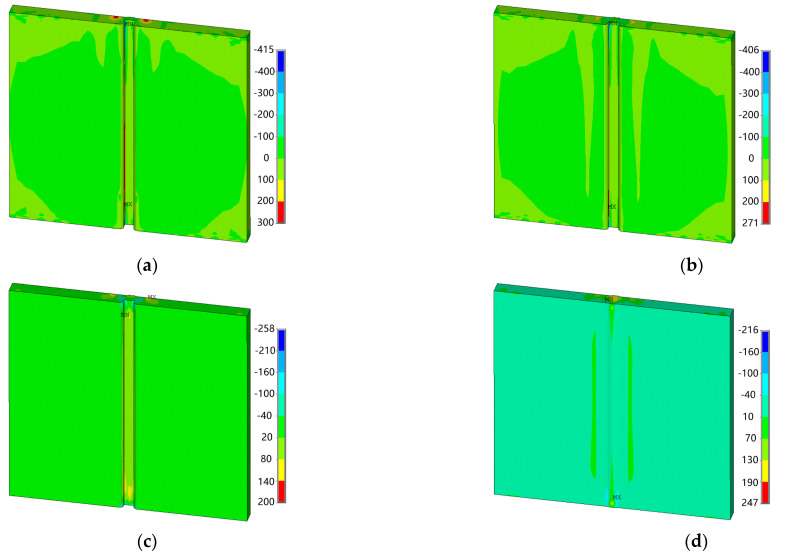
Distribution of the stress in the thickness direction of each weld layer (*f* = 3 Hz, MPa): (**a**) the 1st layer; (**b**) the 2nd layer; (**c**) the 3rd layer; and (**d**) the 4th layer.

Figure 15 presents the distribution of residual stress in the thickness direction for each layer of welds. It can be seen that after the first layer is welded, the residual stress in the thickness direction is concentrated at the weld toe and the starting arc cross section, presenting tensile stress, while the remaining areas on the front side of the test plate show uniformly distributed compressive stress. After the second layer is welded, the residual stress in the thickness direction is concentrated at the weld toe of the tail weld, presenting tensile stress, and the remaining areas on the front side of the test plate still show uniformly distributed compressive stress. After the third layer is welded, the residual stress in the thickness direction is concentrated in the near-seam area on the back side, presenting tensile stress, while the tensile stress at the weld toe on the front side of the weld seam decreases, and the remaining areas on the front side of the test plate still show uniformly distributed compressive stress. After the fourth layer is welded, the residual stress in the thickness direction is concentrated at the cross section of the arc ending, presenting tensile stress, while the compressive stress is concentrated in the near-seam area of the back weld seam. Most areas on the front side of the test plate show uniformly distributed compressive stress; only the head weld seam, tail weld seam, and near-seam area present tensile stress, but the tensile stress values are relatively small.

**Figure 16:**
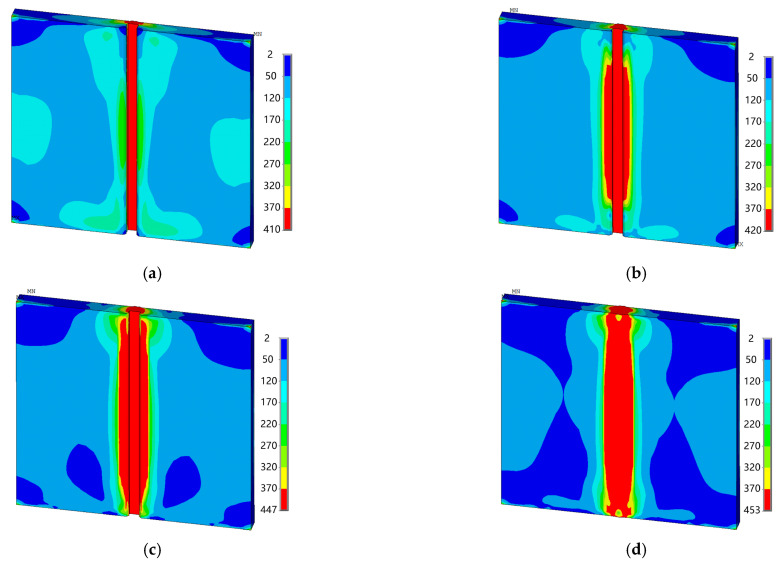
Distribution of von Mises equivalent stress of each weld layer (*f* = 3 Hz, MPa): (**a**) the 1st layer; (**b**) the 2nd layer; (**c**) the 3rd layer; and (**d**) the 4th layer.

Figure 16 shows von Mises equivalent stress distribution cloud maps of each layer of the weld seam. It can be seen that after welding the first layer, the von Mises equivalent stress is mainly concentrated at the weld seam, and the rest is in the near-weld area. The peak value of the equivalent stress is 410 MPa, which is lower than the yield limit of the base metal of 450 MPa. In the area far from the weld, the von Mises equivalent stress is relatively small. After the second layer is welded, the von Mises equivalent stress is mainly concentrated in the weld area and the near-weld area in the middle of the test plate. The peak value of the von Mises equivalent stress is 420 MPa, which is still lower than the yield limit of the base material. In the area far from the weld, the von Mises equivalent stress remains relatively small and is distributed relatively uniformly. After the third layer is welded, the von Mises equivalent stress is mainly concentrated in the weld area and the near-weld area, with the peak value of the von Mises equivalent stress being 447 MPa, slightly lower than the yield limit of the base material. In the area far from the weld, it shows a uniformly distributed low equivalent stress. After the fourth layer is welded, the von Mises equivalent stress is concentrated in the weld area and the near-weld area, with the peak value of the von Mises equivalent stress being 453 MPa, slightly higher than the yield limit of the base material. In the area far from the weld, it is still a uniformly distributed low stress. From this, it can be seen that the von Mises equivalent stress is mainly concentrated in the weld area and the near-weld area. As the welding layers are applied, the peak value of the von Mises equivalent stress gradually increases. The peak values of the first three layers of welds are lower than the yield limit of the base material, while that of the last layer is slightly higher than the yield limit of the base material. In the area far from the weld, the von Mises equivalent stress is relatively small and is distributed relatively uniformly.

**Figure 17:**
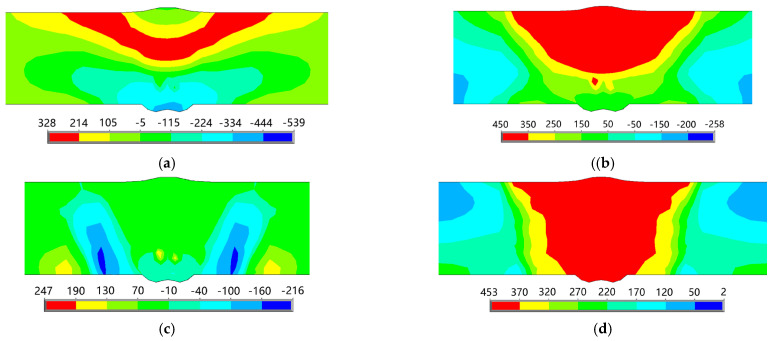
Contours of residual stress at the cross section of weldment of *z* = 150 mm: (**a**) transverse stress; (**b**) longitudinal stress; (**c**) stress in the thickness direction; and (**d**) von Mises equivalent stress.

Figure 17 illustrates the distribution of the welding residual stress components at the central section, as well as the equivalent stress at the weld section. It is evident that both the longitudinal and von Mises equivalent stresses are relatively uniformly distributed within the weld zone and heat-affected zone; however, transverse stress exhibits significant variation in these regions.

### 5.2. Comparison of Distortion

**Figure 18:**
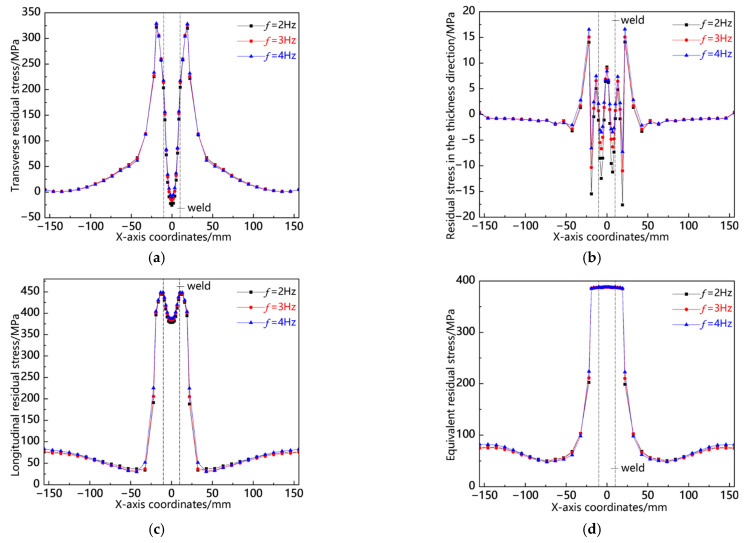
Residual stress distribution curve of L1 sampling line under different swing frequencies: (**a**) transverse stress; (**b**) stress in the thickness direction; (**c**) longitudinal stress; and (**d**) von Mises equivalent stress.

Figure 18 illustrates the distribution curves of residual stress along the L1 sampling line at various swing frequencies. As depicted in Figure 18a, the transverse stress distribution patterns under all three conditions exhibit consistency, primarily concentrated in the near-seam area, where relatively high compressive stresses are observed. The peak values of compressive stress across these conditions are closely aligned, with a slight increase noted at *f* = 4 Hz. In contrast, tensile stress is comparatively low at the weld center, with its maximum value occurring at *f* = 2 Hz. Figure 18b indicates that residual stress in the thickness direction remains generally low and is predominantly localized within both the weld and near-seam areas. Notably, while compressive stress peaks at *f* = 4 Hz, tensile stress reaches its highest value at *f* = 2 Hz. As shown in Figure 18c, the longitudinal stress distributions and peak values across all three conditions are remarkably similar. Furthermore, as illustrated in Figure 18d, the von Mises equivalent stresses under each condition also demonstrate close proximity to one another; specifically, higher stresses occur at *f* = 4 Hz in the near-seam area and workpiece edge, whereas slightly elevated stresses appear at *f* = 2 Hz elsewhere. This analysis reveals that residual stress distribution patterns along the L1 sampling line remain consistent with relatively comparable values.

**Figure 19:**
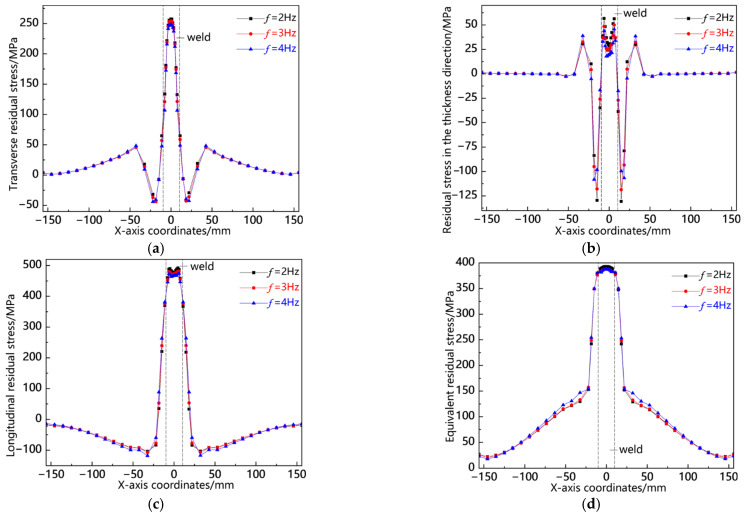
Residual stress distribution curve of L2 sampling line under different swing frequencies: (**a**) transverse stress; (**b**) stress in the thickness direction; (**c**) longitudinal stress; and (**d**) von Mises equivalent stress.

Figure 19 illustrates the distribution curves of residual stress along the L2 sampling line at various swing frequencies. As depicted in Figure 19a, transverse stress is predominantly concentrated in the weld seam, manifesting as compressive stress, with the peak occurring at *f* = 2 Hz; in the near-seam region, relatively low tensile stress is observed, and the stress peaks across all three conditions are approximately equivalent; elsewhere, the stress values gradually approach zero. In Figure 19b, it can be seen that stress in the thickness direction is focused in the near-seam area and appears as tensile stress, with its peak also being highest at *f* = 2 Hz; conversely, within the weld seam area, a comparatively lower compressive stress is noted, again peaking at *f* = 2 Hz. Figure 19c reveals that longitudinal stress concentrates within the weld seam area as a notably high compressive force, with its maximum value recorded at *f* = 2 Hz; meanwhile, some degree of tensile stress exists in the near-seam region where peaks among all three conditions are similar but slightly elevated for f = 4 Hz. Lastly, Figure 19d indicates that the von Mises equivalent stress accumulates primarily within the weld seam area with its peak once more highest at *f* = 2 Hz. The analysis above demonstrates a consistent pattern of residual stress distribution along the L2 sampling line and highlights that a significant peak occurs in this region specifically at *f* = 2 Hz.

**Figure 20:**
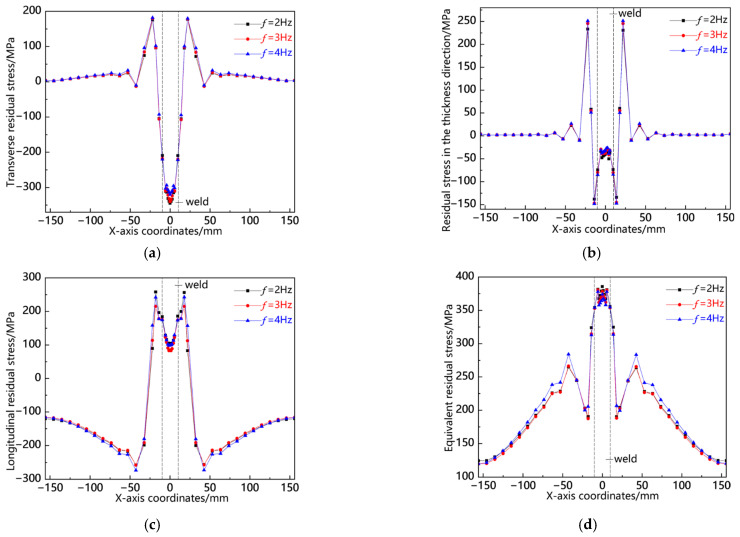
Residual stress distribution curve of L3 sampling line under different swing frequencies: (**a**) transverse stress; (**b**) stress in the thickness direction; (**c**) longitudinal stress; and (**d**) von Mises equivalent stress.

Figure 20 presents the residual stress distribution curves along the L3 sampling line at different swing frequencies. As shown in Figure 20a, the weld zone shows a relatively high transverse tensile stress, with the stress peak being the largest at *f* = 2 Hz; the heat-affected zone shows a certain magnitude of transverse compressive stress, and the stress peaks of the three are basically the same; the transverse stress in other areas is close to zero. As shown in Figure 20b, the residual stress in the thickness direction is concentrated in the heat-affected zone, showing compressive stress, with the stress peak being the largest at *f* = 4 Hz; the weld zone shows a relatively small tensile stress. As shown in Figure 20c, the longitudinal stress is the highest in the heat-affected zone, with the stress peak being the largest at *f* = 2 Hz; the stress in the weld zone is lower than that in the heat-affected zone, and the stress peaks of the three are basically the same; other areas show a certain magnitude of tensile stress. As shown in Figure 20d, the von Mises equivalent stress is concentrated in the weld zone, and the stress peaks of the three are not much different, with that at *f* = 2 Hz being slightly larger. From the above analysis, it can be seen that the residual stress distribution along the L3 sampling line is consistent, with the stress concentrated in the weld zone, and the stress peaks of the three are not much different, with that at *f* = 2 Hz being slightly larger.

**Figure 21:**
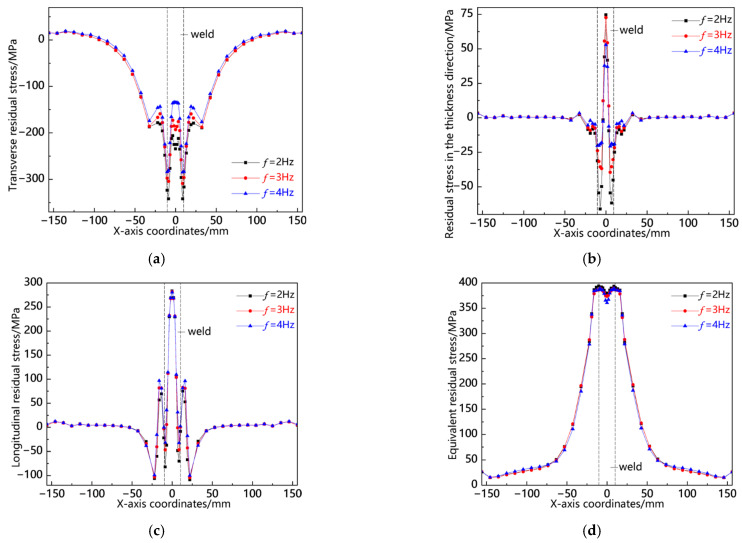
Residual stress distribution curve of L4 sampling line under different swing frequencies: (**a**) transverse stress; (**b**) stress in the thickness direction; (**c**) longitudinal stress; and (**d**) von Mises equivalent stress.

Figure 21 illustrates the distribution curves of residual stress along the L4 sampling line at various swing frequencies. As depicted in Figure 21a, the transverse stress is predominantly concentrated at the weld toe, with a peak stress observed at *f* = 2 Hz, indicating a relatively high tensile stress. The stress within the weld zone is lower than that at the weld toe but still exhibits a notable level of tensile stress, with its peak also occurring at *f* = 2 Hz. In Figure 21b, it can be seen that the thickness direction stresses are primarily localized in the weld zone, where compressive stresses are present at the weld center. The peaks for *f* = 2 Hz and *f* = 3 Hz are comparatively significant; conversely, tensile stresses appear at the weld edge, peaking again at *f* = 2 Hz. Other regions exhibit stresses close to zero. Figure 21c shows that longitudinal stresses concentrate mainly in both the weld zone and adjacent areas, with relatively high compressive stresses noted in these zones. The peaks across all three measurements remain consistent; however, there is a smaller tensile stress observed at the weld toe and minor compressive stresses near this region which rapidly diminish into low tensile values thereafter. Finally, as illustrated in Figure 21d, the von Mises equivalent stress along L4 reaches its maximum within the weld zone; while all three peaks are closely aligned, that corresponding to *f* = 2 Hz is slightly elevated compared to the others. This analysis indicates a consistent pattern of residual stress distribution along L4: notably higher levels exist within the weld zone with an increased peak observed specifically at *f* = 2 Hz.

**Figure 22:**
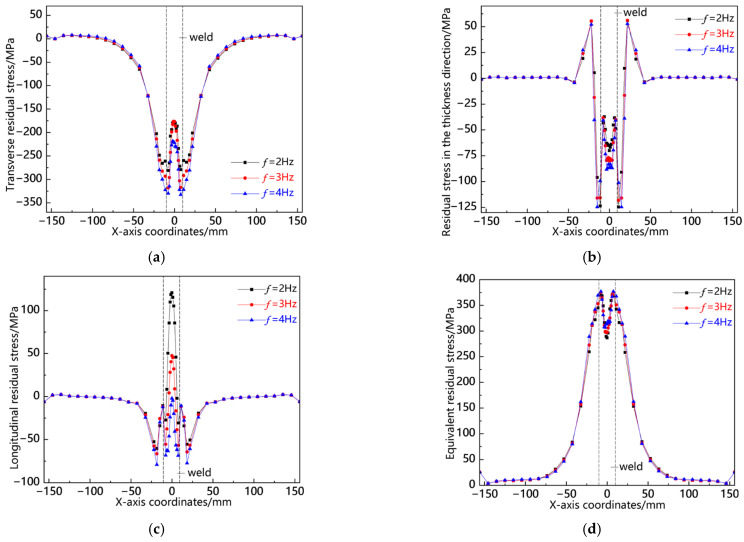
Residual stress distribution curve of L5 sampling line under different swing frequencies: (**a**) transverse stress; (**b**) stress in the thickness direction; (**c**) longitudinal stress; and (**d**) von Mises equivalent stress.

Figure 22 presents the residual stress distribution curves along the L5 sampling line at different shaking frequencies. As shown in Figure 22a, the transverse stress is mainly concentrated at the weld edge, presenting a relatively high tensile stress. Although the weld zone also shows tensile stress, its value is smaller than that near the weld toe. As depicted in Figure 22b, the weld zone and the area near the weld toe exhibit tensile stress, with the stress peak slightly larger at *f* = 4 Hz. The near-seam area shows a relatively small compressive stress, and the stress peaks of the three areas are basically the same. As illustrated in Figure 22c, the longitudinal stress is mainly concentrated in the weld zone and the near-seam area. The weld zone shows compressive stress, with the stress peak being the largest at *f* = 2 Hz. The near-seam area shows tensile stress, with the stress peak being the largest at *f* = 4 Hz. The stress values in other areas are basically the same for the three cases. As shown in Figure 22d, the von Mises equivalent stress is mainly concentrated in the weld zone and the near-seam area, reaching the maximum near the weld toe, and the peak values of the three cases are relatively close. From the above analysis, it can be concluded that the residual stress distribution pattern along L5 is the same. The longitudinal stress peak is the largest at *f* = 2 Hz, and the peak values of the transverse stress, stress in the thickness direction, and von Mises equivalent stress are relatively close.

**Figure 23:**
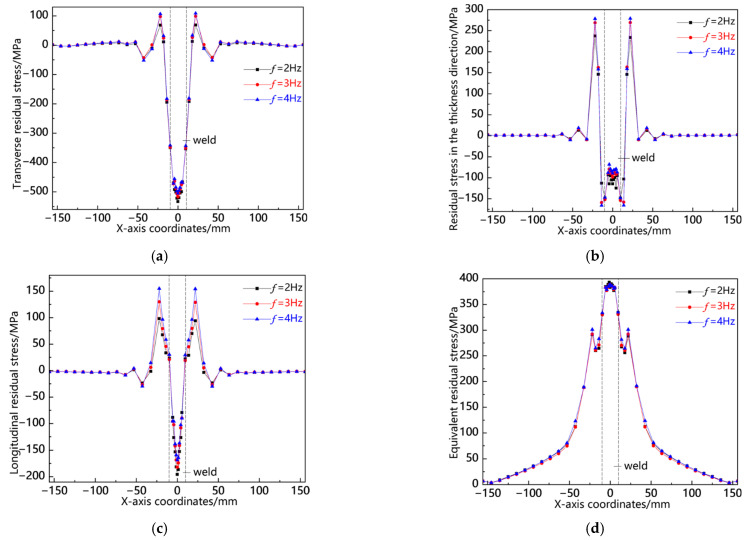
Residual stress distribution curve of L6 sampling line under different swing frequencies: (**a**) transverse stress; (**b**) stress in the thickness direction; (**c**) longitudinal stress; and (**d**) von Mises equivalent stress.

Figure 23 presents the residual stress distribution curves along the L6 sampling line at different swing frequencies. As shown in Figure 23a, the transverse stress is concentrated at the center of the weld seam, presenting a relatively large tensile stress, with the stress peak being the largest at *f* = 2 Hz; the heat-affected zone shows a relatively small compressive stress, with the stress peak being slightly larger at *f* = 4 Hz. As shown in Figure 23b, stress in the thickness direction is concentrated in the heat-affected zone and the weld seam area, with the heat-affected zone showing a relatively high compressive stress and the stress peak being the largest at *f* = 4 Hz; the weld seam area shows a certain magnitude of tensile stress. As shown in Figure 23c, the longitudinal stress is concentrated in the weld seam area and the heat-affected zone, with the weld seam area showing tensile stress and the stress peak being the largest at *f* = 2 Hz; the heat-affected zone shows compressive stress, with the stress peak being the largest at *f* = 4 Hz. As shown in Figure 23d, the von Mises equivalent stress is concentrated in the weld seam area and the heat-affected zone, with the stress peak being reached at the center of the weld seam, and the stress peak being the largest at *f* = 2 Hz. From the above analysis, it can be seen that the residual stress distribution pattern along L6 is the same, with the stress mainly concentrated in the weld seam area and the heat-affected zone and the stress peak being the largest at *f* = 2 Hz.

**Figure 24:**
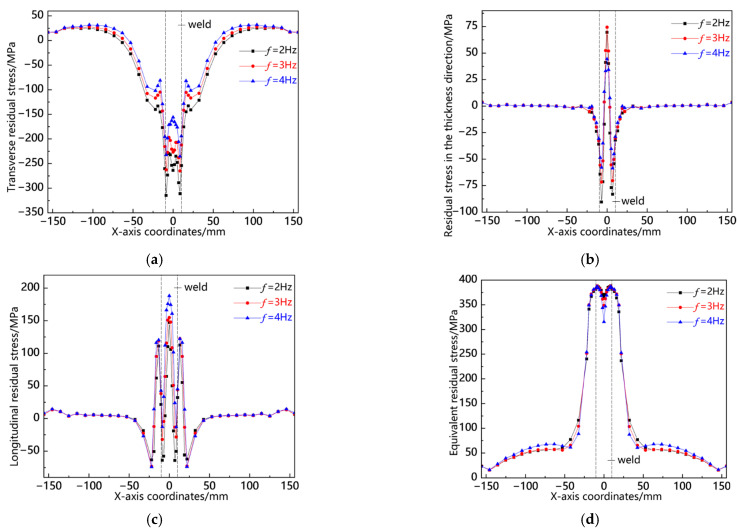
Residual stress distribution curve of L7 sampling line under different swing frequencies: (**a**) transverse stress; (**b**) stress in the thickness direction; (**c**) longitudinal stress; and (**d**) von Mises equivalent stress.

Figure 24 presents the residual stress distribution curves along the L7 sampling line at different swing frequencies. As shown in Figure 24a, the transverse residual stress is concentrated in the weld zone and the heat-affected zone, reaching its peak near the weld toe, with the maximum stress peak at *f* = 2 Hz; the stress in the weld zone is less than that at the weld toe, and the stress is larger at *f* = 2 Hz; the stress in the zone far from the weld is smaller, and it is slightly larger at *f* = 4 Hz. As shown in Figure 24b, the residual stress in the thickness direction is concentrated in the weld zone, with compressive stress at the center of the weld, and the stress is the smallest at *f* = 4 Hz; from the center of the weld to both sides, the stress rapidly decreases until tensile stress is shown, with the maximum tensile stress peak at *f* = 2 Hz; the stress in other areas is smaller, and its value approaches 0. As shown in Figure 24c, the longitudinal stress is concentrated in the weld zone and the heat-affected zone, reaching its peak at the center of the weld, with the maximum stress peak at *f* = 4.0. As shown in Figure 24d, the von Mises equivalent stress is concentrated in the weld zone and the heat-affected zone, reaching its peak at the weld toe, with the stress at the center of the weld being less than that at the weld toe, and the stress is smaller at *f* = 4 Hz. From the above analysis, it can be seen that the distribution pattern of residual stress along L7 is the same, with the stress mainly concentrated in the weld zone and near the weld toe. In the weld zone, the stress is greater at *f* = 2 Hz, while in the zone far from the weld, the stress is greater at *f* = 4 Hz.

**Figure 25:**
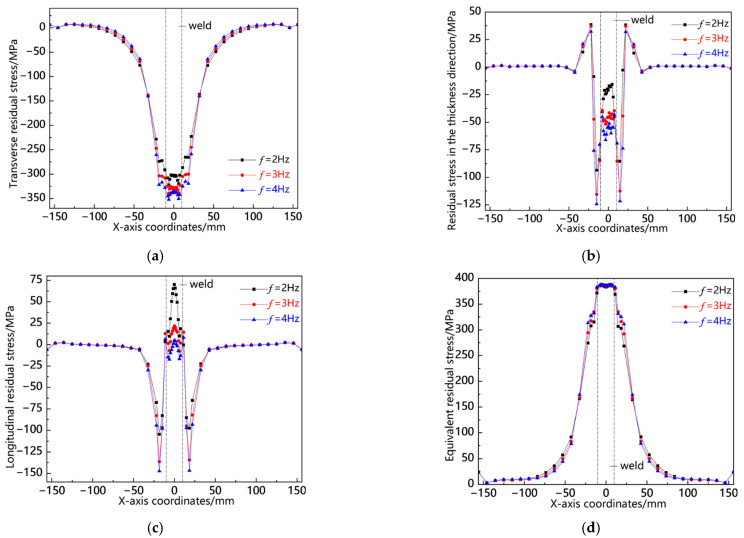
Residual stress distribution curve of L8 sampling line under different swing frequencies: (**a**) transverse stress; (**b**) stress in the thickness direction; (**c**) longitudinal stress; and (**d**) von Mises equivalent stress.

Figure 25 presents the residual stress distribution curves along the L8 sampling line at different swing frequencies. As shown in Figure 25a, the transverse residual stress is mainly concentrated in the weld zone and the area near the weld toe, presenting a relatively high tensile stress, with the stress peak being the largest at *f* = 4 Hz. As depicted in Figure 25b, the residual stress in the thickness direction reaches its maximum near the weld toe, showing tensile stress, with the stress peak being the largest at *f* = 4 Hz; then, the stress rapidly decreases until it becomes compressive stress, with the compressive stress peak being the largest at *f* = 2 Hz. As illustrated in Figure 25c, the longitudinal residual stress is concentrated in the weld zone and the near-weld area, but there are significant differences between the two areas. In the weld zone, it shows compressive stress, with the compressive stress peak being the largest at *f* = 2 Hz; in the near-weld area, it shows tensile stress, with the tensile stress peak being the largest at *f* = 4 Hz. As shown in Figure 25d, the von Mises equivalent stress is concentrated in the weld zone and the near-weld area, and the stress peaks of the three are relatively close. From the above analysis, it can be seen that the residual stress along the L8 sampling line, whether in terms of distribution characteristics or stress magnitude, is relatively close among the three.

**Figure 26:**
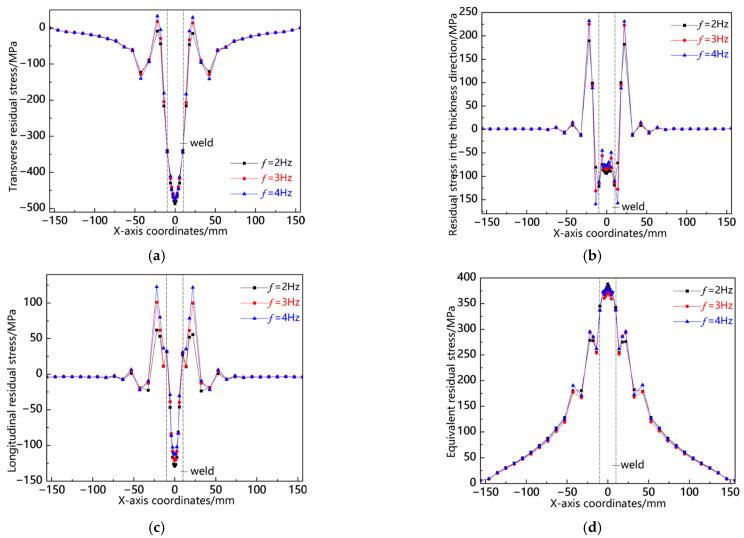
Residual stress distribution curve of L9 sampling line under different swing frequencies: (**a**) transverse stress; (**b**) stress in the thickness direction; (**c**) longitudinal stress; and (**d**) von Mises equivalent stress.

Figure 26 presents the residual stress distribution curves along the L9 sampling line at different swing frequencies. As shown in Figure 26a, the transverse residual stress is concentrated in the weld zone, presenting a relatively high tensile stress, and reaches its peak at the center of the weld. The peak stress is the largest when *f* = 2 Hz. The stresses in the near-seam and far-seam areas are relatively small, and the edge of the workpiece tends to approach 0. As shown in Figure 26b, the residual stress in the thickness direction is concentrated in the near-weld area, presenting tensile stress at the weld toe and relatively high compressive stress in the near-weld area. The peak stress is the largest when *f* = 4 Hz. As shown in Figure 26c, the longitudinal stress is concentrated in the weld zone and the near-weld area, but the stress manifestations in the two zones are different. The weld zone shows longitudinal tensile stress, with the peak tensile stress being the largest when *f* = 2 Hz. The near-weld area shows compressive stress, with the peak compressive stress being the largest when *f* = 4 Hz. As shown in Figure 26d, von Mises equivalent stress is mainly concentrated in the weld zone, reaching its peak at the center of the weld. The peak stress is the largest when *f* = 2 Hz. The stress in the near-weld area is slightly larger when f = 4 Hz. From the above analysis, it can be seen that the distribution pattern of residual stress along L9 is consistent, mainly concentrated in the weld and the near-weld area. The stress in the weld zone is the largest when *f* = 2 Hz, while the stress levels in the near-seam area and the far-seam area alternate.

**Figure 27:**
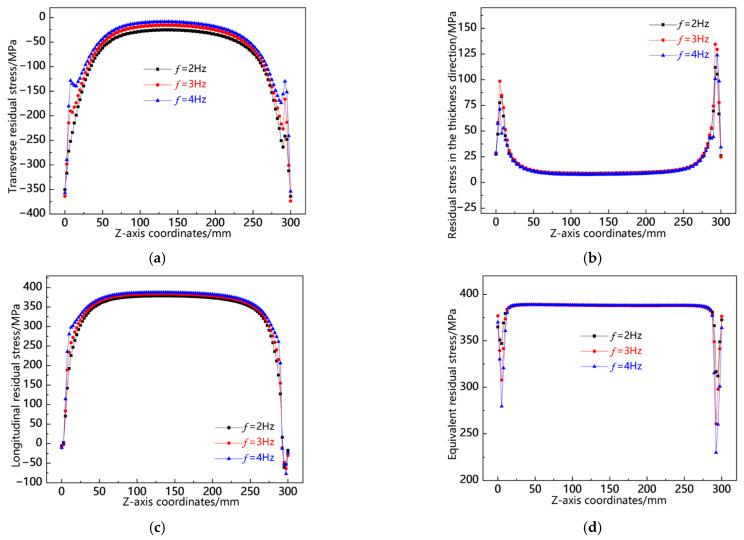
Residual stress distribution curve of L10 sampling line under different swing frequencies: (**a**) transverse stress; (**b**) stress in the thickness direction; (**c**) longitudinal stress; and (**d**) von Mises equivalent stress.

Figure 27 shows the residual stress distribution curves along the L10 sampling line at different swing frequencies. As shown in Figure 27a, the transverse residual stress at the head and tail of the weld is greater than that in the middle of the weld, and the overall transverse stress magnitude is highest at *f* = 2 Hz, followed by *f* = 3 Hz, and lowest at *f* = 4 Hz. As shown in Figure 27b, the residual stress in the thickness direction is also concentrated at the head and tail of the weld, and the stress in the middle of the weld is relatively small. The overall stress magnitude is very close. As shown in Figure 27c, the longitudinal stress is concentrated in the middle of the weld seam, and the values of the three are relatively close. As shown in Figure 27d, in the middle of the weld seam, the von Mises equivalent stresses of the three are almost the same, while at the head and tail of the weld, the equivalent stress at *f* = 4 Hz is the smallest. From the above analysis, it can be seen that along the L10 sampling line, the residual stresses of the three are not significantly different overall, while *f* = 4 Hz has lower transverse residual stresses.

**Figure 28:**
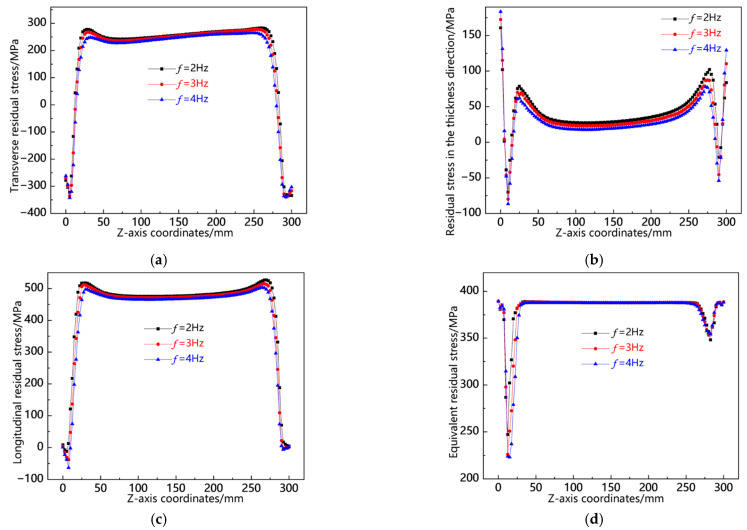
Residual stress distribution curve of L11 sampling line under different swing frequencies: (**a**) transverse stress; (**b**) stress in the thickness direction; (**c**) longitudinal stress; and (**d**) von Mises equivalent stress.

Figure 28 illustrates the distribution curves of residual stress along the L11 sampling line at various shaking frequencies. The data presented in the figure indicate that transverse residual stress, stress in the thickness direction, and longitudinal residual stress all exhibit compressive characteristics. Notably, at a frequency of *f* = 2 Hz, these stresses reach their maximum values; however, the differences among them are relatively minor. Furthermore, the von Mises equivalent stresses for all three types are closely aligned.

**Figure 29:**
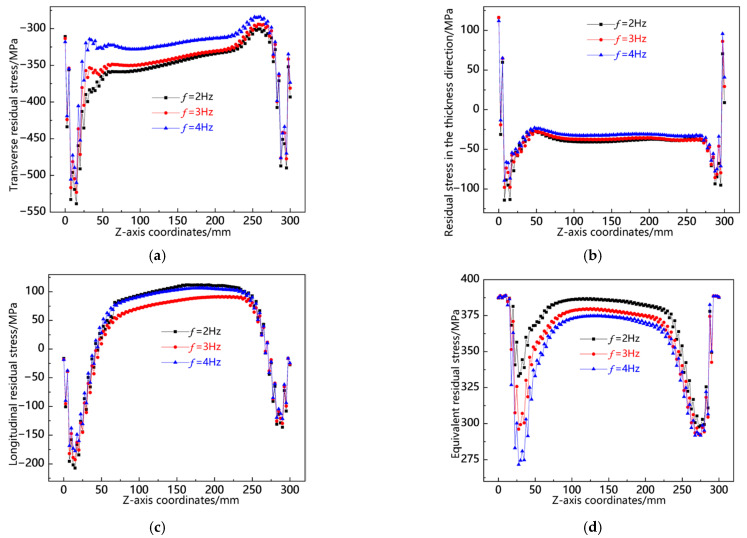
Residual stress distribution curve of L12 sampling line under different swing frequencies: (**a**) transverse stress; (**b**) stress in the thickness direction; (**c**) longitudinal stress; and (**d**) von Mises equivalent stress.

Figure 29 illustrates the distribution curves of residual stress along the L12 sampling line at various swing frequencies. As indicated in Figure 29a, the transverse residual stress is predominantly tensile, with the minimum stress value observed at *f* = 4 Hz and the maximum at *f* = 2 Hz. Figure 29b reveals that both ends of the weld seam exhibit relatively high compressive stress in the thickness direction, while the central region displays a comparatively low tensile stress. The highest stress value occurs at *f* = 2 Hz among all three frequencies; however, variations between them are minimal. In Figure 29c, it can be seen that both ends of the weld seam experience a notable degree of longitudinal tensile stress, with the peak tensile stress recorded at *f* = 2 Hz being significantly higher; conversely, the middle section shows some level of compressive stress. According to Figure 29d, the von Mises equivalent stresses reach their peak at *f* = 2 Hz, followed by *f* = 3 Hz, and then decrease further at *f* = 4 Hz. From this analysis, it can be concluded that along the L12 sampling line, the residual stresses are markedly lower at *f* = 4 Hz compared to those observed at *f* = 2 Hz.

**Figure 30:**
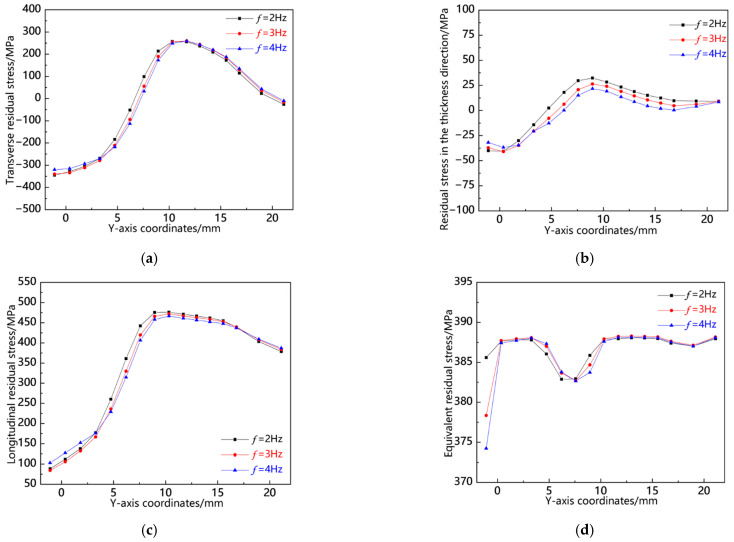
Residual stress distribution curve of L13 sampling line under different swing frequencies: (**a**) transverse stress; (**b**) stress in the thickness direction; (**c**) longitudinal stress; and (**d**) von Mises equivalent stress.

Figure 30 illustrates the distribution curves of residual stress along the L13 sampling line at various swing frequencies. It is evident that, along this sampling line, both transverse and thickness-direction residual stresses transition from tensile to compressive states, peaking around Y = 10 mm. The reason for this is that Y = 10 mm is the middle of the thickness direction of the workpiece, and the middle weld metal is constrained by the upper and lower weld metals, resulting in the highest degree of constraint and higher residual stress. Conversely, longitudinal residual stress exhibits a gradual increase, also reaching its maximum at approximately Y = 10 mm, indicating a relatively high level of residual stress at the center of the weld seam. At different frequencies, it is observed that the residual stress at *f* = 4 Hz is comparatively lower; notably, the equivalent stress values at the weld head are diminished under this frequency condition. Along the L13 sampling line, *f* = 2 Hz demonstrates reduced transverse residual stress, while *f* = 4 Hz shows decreased thickness-directional, longitudinal, and von Mises equivalent stresses overall. Therefore, it can be concluded that among these conditions tested, *f* = 4 Hz yields the lowest overall residual stress.

## 6. Conclusions

The conclusions of this study are as follows:

(1) Considering the characteristics of the welding process, the joint geometry, and the moving trajectory of the heat source in a comprehensive manner, a three-dimensional finite element numerical analysis model for residual stress in swing-arc narrow-gap GMA welding has been established based on elastic–plastic theory. The distribution characteristics of residual stress in Q370qE steel during swing-arc narrow-gap GMA welding were quantitatively investigated, and the effect of swing frequency on the distribution of welding residual stress was analyzed. The residual stress within the workpiece was measured using the blind hole method and compared with computational results to validate the accuracy of the model. Through the simulation of welding residual stress results in this paper, the swing-arc GMA welding parameters can be optimized to reduce residual stress and ensure that the welding process and product quality meet industry standards and regulatory requirements. This can also predict the possible deformation and cracking during the welding process, helping to take measures in advance to avoid structural failure.
(2) Along the direction of the weld seam, residual stress is primarily concentrated in the middle region, exhibiting relatively lower levels at both the starting and ending points of the weld. Perpendicular to this direction, residual stress is predominantly found within the weld zone and heat-affected zone, with diminished levels observed in more distant areas. In terms of the thickness direction, the residual stress is minimal at the bottom of the weld seam, followed by a moderate level at its top, while it peaks in intensity at the center of the weld seam.
(3) Under the swing frequencies of 2 Hz, 3 Hz, and 4 Hz, the distribution patterns of residual stress along each sampling line exhibit a consistent trend. The residual stress is predominantly concentrated in both the weld zone and the heat-affected zone, while it remains relatively low in areas distant from the weld.
(4) The residual stress within the weld is minimized at a frequency of 4 Hz. In the vicinity of the weld, the magnitudes of residual stress exhibit alternating patterns. Conversely, in the distal weld zone, the variations in residual stress magnitudes among the three conditions are relatively minor. Although the heat input and constraint conditions are the same, the sidewall dwell time under different swing frequencies is 60 ms. As the swing frequency increases, the distribution of the arc heat on the sidewall and in the middle of the weld bead becomes more uniform, resulting in lower residual stress.

## Data Availability

The original contributions presented in this study are included in this article. Further inquiries can be directed to the corresponding author.
